# Effects of intermittent fasting combined with resistance training on training adaptations: an exploratory multilevel meta-analysis

**DOI:** 10.3389/fnut.2026.1879031

**Published:** 2026-06-24

**Authors:** Mengyuan Zhang, Sha Wang, Sudawan Wutichat, Theeranan Tanphanich, Hansen Li, Xing Zhang

**Affiliations:** 1 Physical Education College, Xichang University, Xichang, China; 2 Faculty of Education and Development Sciences, Kasetsart University Kamphaeng Saen Campus, Nakhon Pathom, Thailand; 3 School of Physical Education, Sichuan Agricultural University, Ya'an, China; 4 Department of Physical Education and Sports, Faculty of Sport Sciences, University of Granada, Granada, Spain

**Keywords:** muscle strength, power, resistance training, strength endurance, velocity

## Abstract

**Background:**

Intermittent fasting (IF) is increasingly combined with resistance training (RT), yet its effects on RT-specific adaptations remain unclear because previous reviews have mainly focused on body composition and metabolic outcomes.

**Objective:**

This exploratory multilevel meta-analysis compared IF combined with RT versus RT alone for training adaptations, including maximal strength, local muscular endurance, hypertrophy-related outcomes, and explosive/high-speed performance.

**Methods:**

PubMed, Web of Science, and SPORTDiscus were searched to 10 March 2026. Eligible studies were peer-reviewed English-language randomized parallel-group or crossover interventions comparing IF plus RT with RT alone for at least 4 weeks in generally healthy participants. Risk of bias was assessed using the Cochrane Risk of Bias 2 tool. Hedges’ g values based on between-group change scores were pooled using multilevel random-effects models with cluster-robust variance estimation.

**Results:**

Eleven studies involving 303 participants were included. Participants were mostly young adults, with varied training status and body-composition profiles. Interventions lasted 4 weeks to 12 months, most commonly used time-restricted eating/feeding, and generally matched RT programs between arms. IF plus RT produced broadly similar training adaptations to RT alone overall (*g* = 0.09, 95% CI −0.23 to 0.40). No clear differences were observed for maximal strength, local muscular endurance, site-specific hypertrophy-related outcomes, explosive/high-speed performance, or body-region subgroups.

**Conclusion:**

Current limited evidence does not indicate that IF combined with RT clearly compromises chronic RT-related adaptations when RT and nutritional support are reasonably maintained. Further well-controlled research is warranted.

## 1 Introduction

Resistance training (RT) is widely recognized as a cornerstone intervention for improving muscular strength, skeletal muscle hypertrophy, and physical performance (1–6). It is also associated with important health benefits, including improvements in body composition (7), bone mineral density (8), functional capacity (9), and mental health outcomes such as health-related quality of life and depressive symptoms (9). Accordingly, RT is recommended across both athletic and general populations. The magnitude and quality of RT-induced adaptations are influenced, at least in part, by nutritional factors; therefore, dietary strategies related to protein intake, energy restriction, and nutrient timing are frequently implemented alongside RT to support training performance, recovery, and body composition goals (10, 11). In recent years, intermittent fasting (IF) has gained increasing attention as another practical dietary approach that may be combined with RT (12, 13).

Intermittent fasting refers to a broad class of eating patterns characterized by recurrent periods of little or no energy intake interspersed with periods of habitual or unrestricted feeding (12, 14). Common IF subtypes include alternate-day fasting, periodic or whole-day fasting, and time-restricted eating/feeding, which confines daily food intake to a defined eating window and thereby creates a prolonged fasting period (12, 14). Although these regimens differ in structure, they share the feature of extending fasting beyond a typical overnight fast and, in some cases, reducing feeding opportunities and spontaneous energy intake (12, 14). From an RT perspective, IF may influence adaptations not only by altering total energy intake, but also by modifying meal timing, protein distribution, feeding opportunities, and the temporal relationship between nutrient intake and exercise (15–18). These factors could theoretically affect recovery and chronic training adaptations, contributing to growing interest in IF among strength and conditioning practitioners.

To date, a growing number of experimental studies have examined IF combined with RT, but findings remain inconsistent (19–22). For example, Moro et al. (19) conducted an 8-week randomized trial in resistance-trained men and reported that a eucaloric 16:8 time-restricted feeding regimen reduced fat mass while maintaining fat-free mass and maximal strength. In contrast, Stratton et al. (20) examined a 4-week hypocaloric 16:8 time-restricted feeding intervention combined with supervised full-body RT in recreationally active young men and found no clear advantage over a non-fasting comparator for body composition or muscle performance. These mixed findings highlight the need for a focused synthesis of the available evidence to clarify whether IF combined with RT affects RT-related training adaptations.

To address the limitations of individual experimental studies, several systematic reviews and meta-analyses have synthesized the effects of IF combined with RT (23–25). However, previous syntheses have primarily focused on body composition and metabolic health, generally suggesting that IF combined with RT may reduce body mass and fat mass while largely preserving fat-free mass (23–25). Although informative, these outcomes do not fully capture RT-specific training adaptations. Outcomes such as maximal strength, local muscular endurance, site-specific hypertrophy-related measures, and explosive or high-speed performance have received comparatively less dedicated synthesis. Therefore, this exploratory systematic review and multilevel meta-analysis aimed to compare IF combined with RT versus RT without IF in generally healthy participants after interventions lasting at least 4 weeks, focusing on maximal strength, local muscular endurance, site-specific hypertrophy-related outcomes, and explosive/high-speed performance.

## 2 Methods

### 2.1 Protocol registration and search strategy

This systematic review and multilevel meta-analysis was conducted in accordance with the Cochrane Handbook for Systematic Reviews of Interventions and reported following the PRISMA 2020 statement (26). The protocol was prospectively registered in PROSPERO (CRD420261347819).

PubMed (MEDLINE), Web of Science Core Collection, and SPORTDiscus (EBSCOhost) were searched from database inception to 10 March 2026. Search strategies were adapted to the syntax and indexing features of each database and combined free-text terms and, where applicable, controlled vocabulary related to intermittent fasting and resistance training. To maximize search sensitivity, no population, outcome, or date restrictions were applied. The full search strategies for each database are provided in Supplementary Table 1. In addition, backward citation searching was conducted by screening the reference lists of included studies and relevant reviews. Google Scholar was used as a supplementary tool during this process to locate and verify potentially eligible records identified through citation searching. All records were imported into and managed using EndNote 21 (Clarivate Analytics).

### 2.2 Eligibility criteria

Eligibility criteria were defined using the PICOS framework.

Population: Generally healthy participants without severe injuries or medical conditions that would contraindicate or substantially interfere with participation in RT and/or IF interventions.

Intervention: IF combined with an RT program.

Comparison: A comparator condition involving RT without IF.

Outcomes: Eligible outcomes were RT-related training adaptations, including maximal strength, local muscular endurance, site-specific hypertrophy-related outcomes, and explosive/high-speed performance.

Study design: We included original full-text, peer-reviewed English-language intervention studies, including randomized parallel-group trials and randomized crossover trials, in which both groups performed RT and differed primarily in the IF condition (27, 28). Interventions were required to last at least 4 weeks (29). Studies without an RT comparator, non-original full-text journal articles, or non-English publications were excluded.

### 2.3 Study selection and data extraction

All records were imported into EndNote 21, and duplicates were removed through automated matching, with manual checking performed when necessary. Two reviewers (XZ and HSL) independently screened titles and abstracts against the predefined eligibility criteria. Full texts of potentially relevant articles were then retrieved and independently assessed by the same reviewers. Disagreements were resolved through discussion, and a third reviewer (MYZ) was consulted when required.

Data from each included study were extracted using a standardized Excel spreadsheet that was pilot-tested and refined before formal extraction. Extracted variables included study identification, study design, participant characteristics, intervention and RT prescription, comparator characteristics, and outcome data, including assessment time points, units, means, standard deviations (SDs), and change scores where available. When outcome data were available only in graphical format, values were digitized using GetData Graph Digitizer v2.26 (GetData Software Pty Ltd., Kogarah, NSW, Australia). Two reviewers (MYZ and HSL) independently performed data extraction, with disagreements resolved through discussion or consultation with a third reviewer (XZ).

### 2.4 Assessment of bias and evidence quality

Risk of bias was assessed independently by two reviewers (XZ and HSL) using the Cochrane Risk of Bias 2 (RoB 2) tool for randomized trials (30). The assessed domains included the randomization process, deviations from intended interventions, missing outcome data, outcome measurement, and selection of the reported result. Each domain and the overall risk of bias were judged as low risk, some concerns, or high risk according to RoB 2 guidance. Disagreements were resolved through discussion, with a third reviewer (MYZ) consulted when necessary.

The certainty of evidence for the primary outcome categories was evaluated using the Grading of Recommendations Assessment, Development and Evaluation (GRADE) approach (31). Evidence from randomized trials was initially rated as high and downgraded, when appropriate, for risk of bias, inconsistency, indirectness, imprecision, and publication bias. Final certainty was classified as high, moderate, low, or very low. Because the number of independent study clusters was insufficient, publication bias was not formally assessed and was judged cautiously based on the available evidence and overall pattern of findings.

### 2.5 Statistical analysis

All meta-analytic procedures were conducted in R using the metafor and clubSandwich packages. Effect sizes were calculated as Hedges’ g based on between-group differences in change scores, with positive values indicating more favorable effects of IF combined with RT relative to RT alone. When change-score standard deviations were not reported, they were estimated from baseline and post-intervention standard deviations using an assumed pre-post correlation of *r* = 0.70. Standard errors were converted to standard deviations when necessary. Sensitivity analyses were conducted using alternative assumed correlations (*r* = 0.30, 0.50, 0.70, and 0.90).

Because several studies contributed multiple effect sizes, pooled estimates were calculated using multilevel random-effects models, with effect sizes nested within study clusters to account for statistical dependence (32). Variance components were estimated using restricted maximum likelihood (REML) (33). Cluster-robust variance estimation (CR2) with Satterthwaite-adjusted degrees of freedom was applied to improve inference given the limited number of independent study clusters (34, 35).

The primary outcomes were maximal strength and local muscular endurance. Site-specific hypertrophy-related outcomes were treated as secondary outcomes, whereas explosive/high-speed performance was analyzed separately as an exploratory outcome because it was less consistently reported and showed greater conceptual and measurement heterogeneity. Pooled effects were estimated overall and, where appropriate, by outcome category and/or body region.

Sensitivity analyses for the primary and secondary outcomes included restricting analyses to high-confidence extracted data, excluding shared-control comparisons, and varying the assumed pre-post correlation. Heterogeneity was examined using variance components from the multilevel models and approximate multilevel I^2^ values, which were interpreted as descriptive heterogeneity indices rather than conventional single-level I^2^ statistics (36, 37). Small-study effects were not formally assessed because the number of independent study clusters was below the recommended minimum for reliable funnel plot asymmetry or Egger-type tests (38, 39). A two-sided *p* < 0.05 was considered statistically significant.

## 3 Results

### 3.1 Study selection

The study selection process is summarized in Supplementary Figure 1. The database search identified 5,144 records, and 111 duplicates were removed. After title and abstract screening, 37 reports, including one additional record identified through Google Scholar, were retrieved and assessed for eligibility. Of these, 26 were excluded because the intervention was not IF combined with RT (*n* = 21), the comparator was not RT alone (*n* = 3), the report was not a journal article (*n* = 1), or the article was not published in English (*n* = 1). Ultimately, 11 studies were included in the review.

### 3.2 Study characteristics

A total of 11 studies involving 303 participants were included. Most participants were young adults, although training status and body composition varied across studies. All included studies used chronic interventions, and most lasted 8 weeks. One study adopted a crossover design with 4-week intervention periods separated by a 2-week washout (21), whereas another study lasted 12 months (40). In all studies, the comparator condition involved RT combined with a non-fasting or habitual dietary pattern, whereas the experimental condition combined RT with a fasting-related dietary strategy, most commonly time-restricted eating/feeding. RT was typically performed 3 sessions per week, and the training program was generally matched between comparison arms, with the dietary strategy representing the principal between-condition difference in most studies. Detailed study characteristics are presented in Supplementary Table 2.

### 3.3 Risk of bias assessment

The risk-of-bias assessment is summarized in Supplementary Figure 2. Overall, four studies were judged at high risk of bias (15, 20, 40, 41), and the remaining seven studies were rated as having some concerns; no study was judged overall at low risk. For the randomization process, only one study was rated as low risk (42), while the others had some concerns due to insufficient reporting of randomization procedures. High risk of bias was most frequently related to deviations from intended interventions (15, 20, 41) and missing outcome data (15, 20, 40, 41). Most studies were judged at low risk for outcome measurement, although two had some concerns (22, 43). For selection of the reported result, three studies were rated as low risk (15, 43, 44), whereas the others had some concerns.

### 3.4 Exploratory multilevel meta-analysis

**Figure 1:**
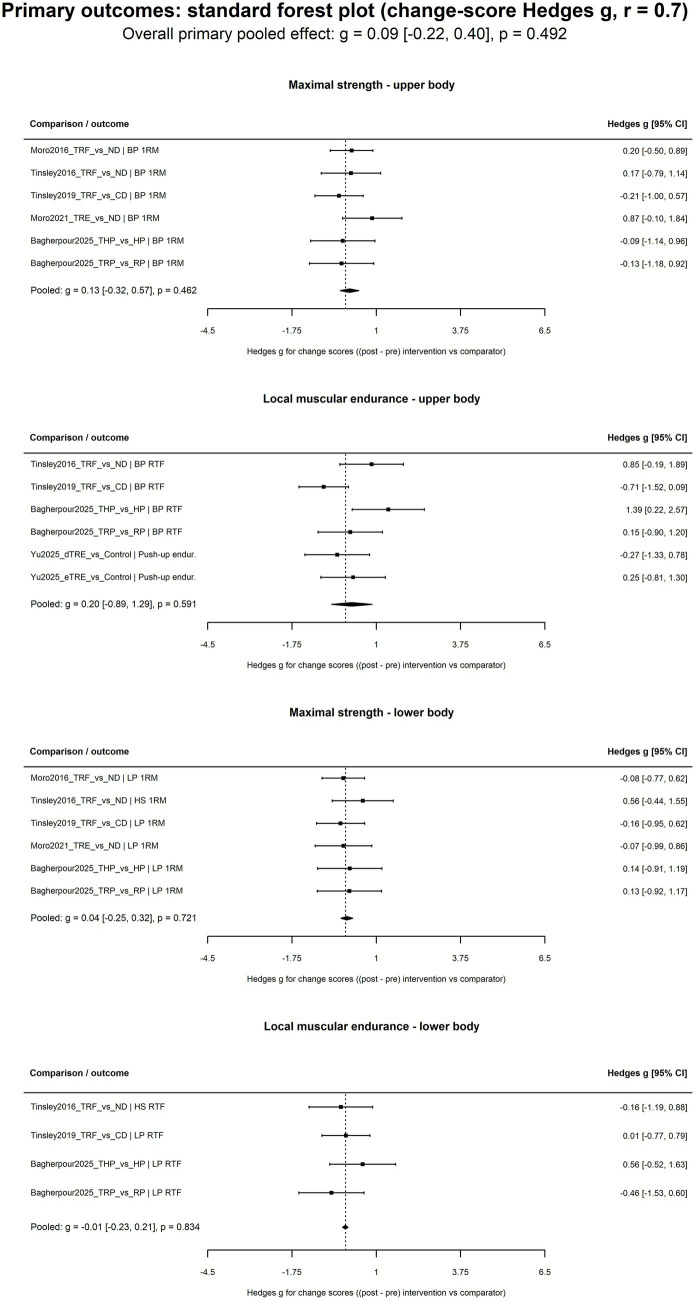
Forest plot of the change-score meta-analysis for primary outcomes comparing IF/time-restricted eating plus resistance training (RT) with RT alone. TRF, time-restricted feeding; TRE, time-restricted eating; dTRE, delayed time-restricted eating; eTRE, early time-restricted eating; ND, normal diet; CD, control diet; FED, habitual or non-time-restricted feeding comparator; HP, high protein; RP, regular protein; THP, time-restricted eating plus high protein; TRP, time-restricted eating plus regular protein; BP, bench press; LP, leg press; HS, hip sled; 1RM, one-repetition maximum; RTF, repetitions to failure.

The exploratory multilevel meta-analysis showed that IF combined with RT produced broadly similar RT-related adaptations compared with RT alone. For the primary outcomes, the pooled effect was small and not statistically significant (*g* = 0.09, 95% CI −0.23 to 0.40, *p* = 0.492; Figure 1), indicating no clear overall difference between conditions. Category-specific analyses also showed no significant differences for maximal strength (*g* = 0.08, 95% CI −0.18 to 0.35, *p* = 0.424; certainty of evidence: moderate) or local muscular endurance (*g* = 0.10, 95% CI −0.55 to 0.75, *p* = 0.634; certainty of evidence: very low). Subgroup analyses by body region were consistent with the main findings, with no clear differences for either upper- or lower-body strength and muscular endurance outcomes.

**Figure 2:**
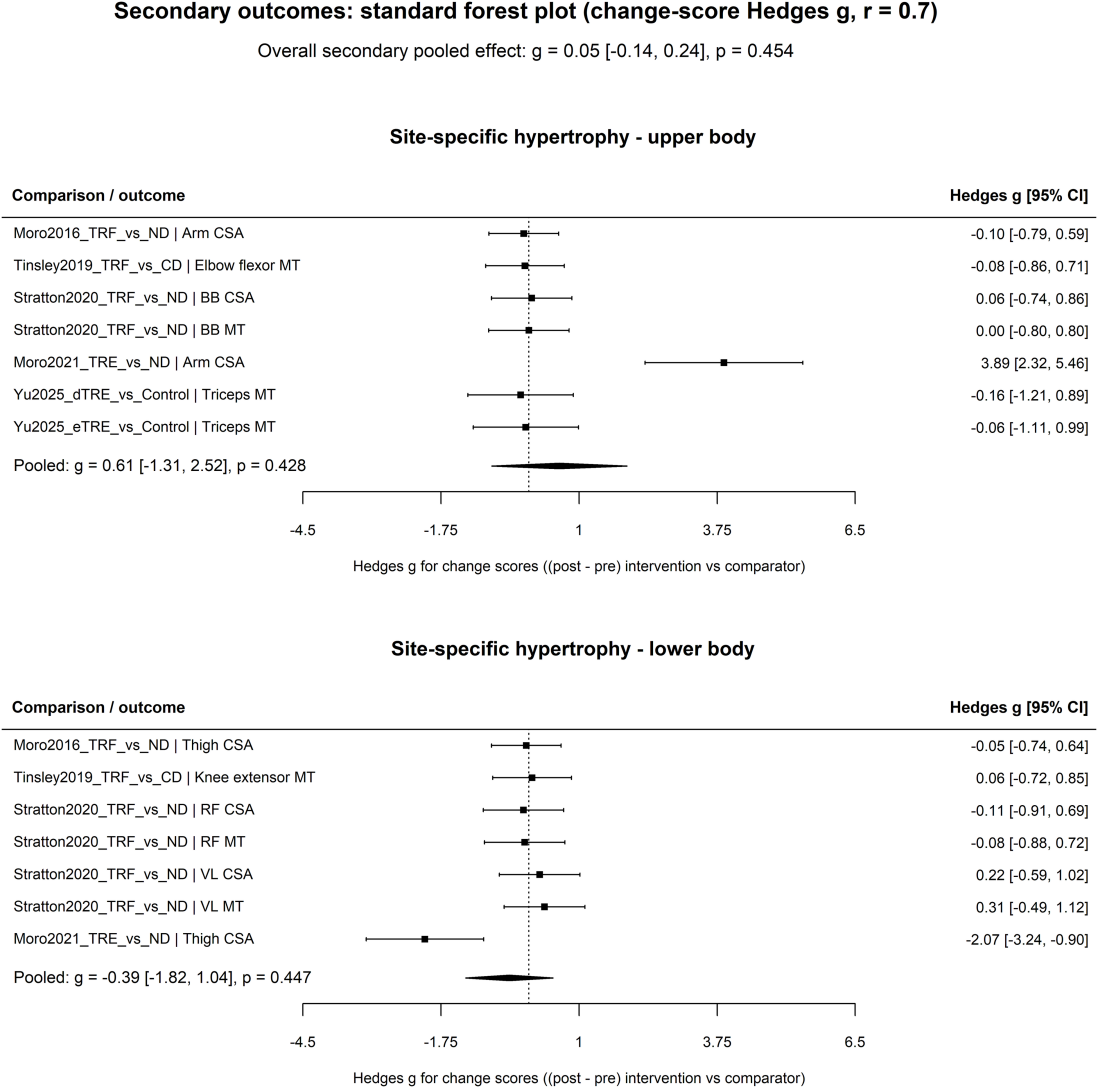
Forest plot of the change-score meta-analysis for secondary outcomes comparing IF/time-restricted eating plus resistance training (RT) with RT alone. TRF, time-restricted feeding; TRE, time-restricted eating; dTRE, delayed time-restricted eating; eTRE, early time-restricted eating; ND, normal diet; CD, control diet; FED, habitual or non-time-restricted feeding comparator; Arm CSA, arm cross-sectional area; BB, biceps brachii; MT, muscle thickness; RF, rectus femoris; VL, vastus lateralis.

For secondary outcomes, IF combined with RT did not produce a significant difference in site-specific hypertrophy-related outcomes compared with RT alone (*g* = 0.05, 95% CI −0.14 to 0.24, *p* = 0.454; Figure 2). Body-region analyses likewise showed no clear differences for upper- or lower-body hypertrophy-related outcomes. Similarly, the exploratory analysis of explosive/high-speed performance showed no significant between-condition difference overall (*g* = 0.03, 95% CI −0.70 to 0.75, *p* = 0.827), with consistent findings for both upper- and lower-body outcomes.

Sensitivity analyses generally supported the robustness of the main findings. For the primary outcomes, excluding shared-control comparisons yielded a similar pooled estimate (*g* = 0.10, 95% CI −0.26 to 0.46, *p* = 0.481), and varying the assumed pre-post correlation produced consistently small, non-significant effects (g = 0.05 to 0.16). Although the high-confidence-only analysis showed a small positive effect (*g* = 0.19, 95% CI 0.01 to 0.38, *p* = 0.042), this estimate was based on a reduced subset of effects and was not consistent with the main analysis or other sensitivity analyses. For secondary hypertrophy-related outcomes, restricting the analysis to high-confidence data (*g* = 0.08, 95% CI −0.27 to 0.43, *p* = 0.447), excluding shared-control comparisons (*g* = 0.09, 95% CI −0.22 to 0.41, *p* = 0.358), and varying the assumed pre-post correlation (*g* = 0.02 to 0.08) did not materially change the interpretation. Approximate heterogeneity was low for primary outcomes (*I*^2^ ≈ 4.9%) but higher for secondary hypertrophy-related outcomes (*I*^2^ ≈ 67.1%), and secondary heterogeneity was not explained solely by extraction confidence or shared-control comparisons. Egger-type regression tests were not performed because the number of independent study clusters was insufficient for reliable inference. The studies and comparisons retained or excluded in each sensitivity analysis are summarized in Supplementary Table 3.

## 4 Discussion

This systematic review and multilevel meta-analysis provides a preliminary quantitative synthesis of the effects of intermittent fasting (IF) combined with resistance training (RT) on RT-related adaptations. Overall, the available evidence did not show clear beneficial or detrimental effects compared with RT alone on maximal strength, local muscular endurance, site-specific hypertrophy-related outcomes, or exploratory explosive/high-speed performance. However, given the small evidence base, short intervention durations, and methodological concerns, these findings should be interpreted cautiously and should not be considered definitive evidence of equivalence between conditions.

### 4.1 Effects on strength, muscular endurance, and hypertrophy-related adaptations

The primary analyses did not show clear evidence that IF combined with RT altered maximal strength or local muscular endurance adaptations compared with RT performed with non-fasting dietary patterns. Similar patterns were observed when upper- and lower-body outcomes were examined separately. One possible explanation is that these adaptations are driven primarily by the RT stimulus itself, including training specificity, progressive overload, sufficient loading, and accumulated training volume, whereas meal timing may exert only a modest additional influence when total daily energy and protein intake are reasonably adequate (1, 2, 18, 45). In many included studies, RT prescriptions were matched between conditions, and energy intake, protein intake, or both were at least partially controlled. Under such circumstances, restricting the daily feeding window may not be sufficient to meaningfully influence neuromuscular adaptation, particularly over the short-to-medium intervention periods examined in most studies. However, as summarized in Supplementary Table 4, reporting of key nutritional factors, particularly daily protein and carbohydrate intake, was incomplete in some studies. This limits the ability to isolate the independent effect of IF from broader dietary factors.

Similarly, IF combined with RT did not show clear evidence of affecting site-specific hypertrophy-related outcomes relative to RT alone. This finding is physiologically plausible because muscle hypertrophy is determined mainly by the cumulative RT stimulus and the broader nutritional environment, especially adequate energy and protein intake, rather than by meal timing alone (2, 10, 11, 45). When daily protein intake can still be achieved within a restricted eating window, IF may not substantially alter the anabolic conditions required for hypertrophy. However, these findings should be interpreted cautiously. The certainty of evidence for local muscular endurance was very low, the pooled estimates for some outcomes were imprecise, and most interventions lasted only 4–8 weeks. Therefore, the current evidence should be viewed as insufficient to demonstrate a clear effect rather than as strong evidence that no effect exists. Longer-term, well-controlled trials are needed before firm conclusions can be drawn regarding the effects of IF combined with RT on strength, muscular endurance, and hypertrophy-related adaptations.

### 4.2 Exploratory explosive/high-speed performance outcomes

The exploratory analysis did not show clear evidence that IF combined with RT affected explosive/high-speed performance. However, this domain requires particular caution because it was synthesized from a heterogeneous set of indicators, including jump-, force-, and ballistic upper-body performance measures. Although the multilevel meta-analytic approach accounted for statistical dependence among effect sizes from the same study, it cannot fully address the conceptual heterogeneity introduced by pooling distinct performance indicators. Accordingly, the current pooled estimate should not be interpreted as evidence that all explosive or high-speed performance outcomes respond similarly to IF. Future studies and evidence syntheses should examine specific indicators separately once a sufficient evidence base becomes available.

### 4.3 Strengths and limitations

This review has several strengths, including its specific focus on RT-related training adaptations rather than body composition or metabolic outcomes alone, its use of multilevel meta-analysis to account for dependent effect sizes, and its application of risk-of-bias and GRADE assessments to support cautious interpretation. By providing preliminary quantitative estimates for maximal strength, local muscular endurance, site-specific hypertrophy-related outcomes, and exploratory explosive/high-speed performance, this review may also inform future trial planning, sample-size estimation, outcome selection, and plausible effect-size parameters or prior distributions for future Bayesian or hierarchical evidence syntheses.

Several limitations should be acknowledged. First, the evidence base was small, with only 11 included studies, which reduced precision, limited subgroup analyses, and precluded reliable formal assessment of small-study effects. Second, most interventions lasted only 4–8 weeks, with only one study extending to 12 months (40), so the findings mainly reflect short- to medium-term adaptations. Third, samples were dominated by young adults, while training status, sex composition, and body-composition profiles varied across studies, limiting generalizability. Fourth, methodological quality was a concern because no study was judged overall at low risk of bias. Finally, explosive/high-speed performance was synthesized from a small number of heterogeneous indicators, which may have introduced conceptual heterogeneity despite the use of a multilevel approach. Future studies should use longer interventions, clearer reporting of IF protocols and nutritional intake, and more consistent outcome selection.

## 5 Conclusion

The current systematic review and multilevel meta-analysis compared the effects of IF combined with RT versus RT alone on training adaptation outcomes. Overall, the available evidence did not show clear beneficial or detrimental effects of IF combined with RT on maximal strength, local muscular endurance, site-specific hypertrophy-related outcomes, or exploratory explosive/high-speed performance. From a practical perspective, these findings suggest that IF may be compatible with RT-related adaptations when the overall training stimulus and nutritional support are adequately maintained. However, given the limited evidence base, short intervention durations, and methodological concerns in the included studies, these findings should be interpreted cautiously and should not be considered definitive evidence of equivalence between conditions.
