# Supplementary material for: Effects of intermittent fasting combined with resistance training on training adaptations: an exploratory multilevel meta-analysis
Source: Front Nutr. 2026 Jun 24;13:1879031. doi: 10.3389/fnut.2026.1879031 (PMC13341873; doi:10.3389/fnut.2026.1879031)
Supplement: Supplementary file 1 [file Supplementary_file_1.DOCX]

Supplementary Material

# Supplementary Figures and Tables

## Supplementary Figures


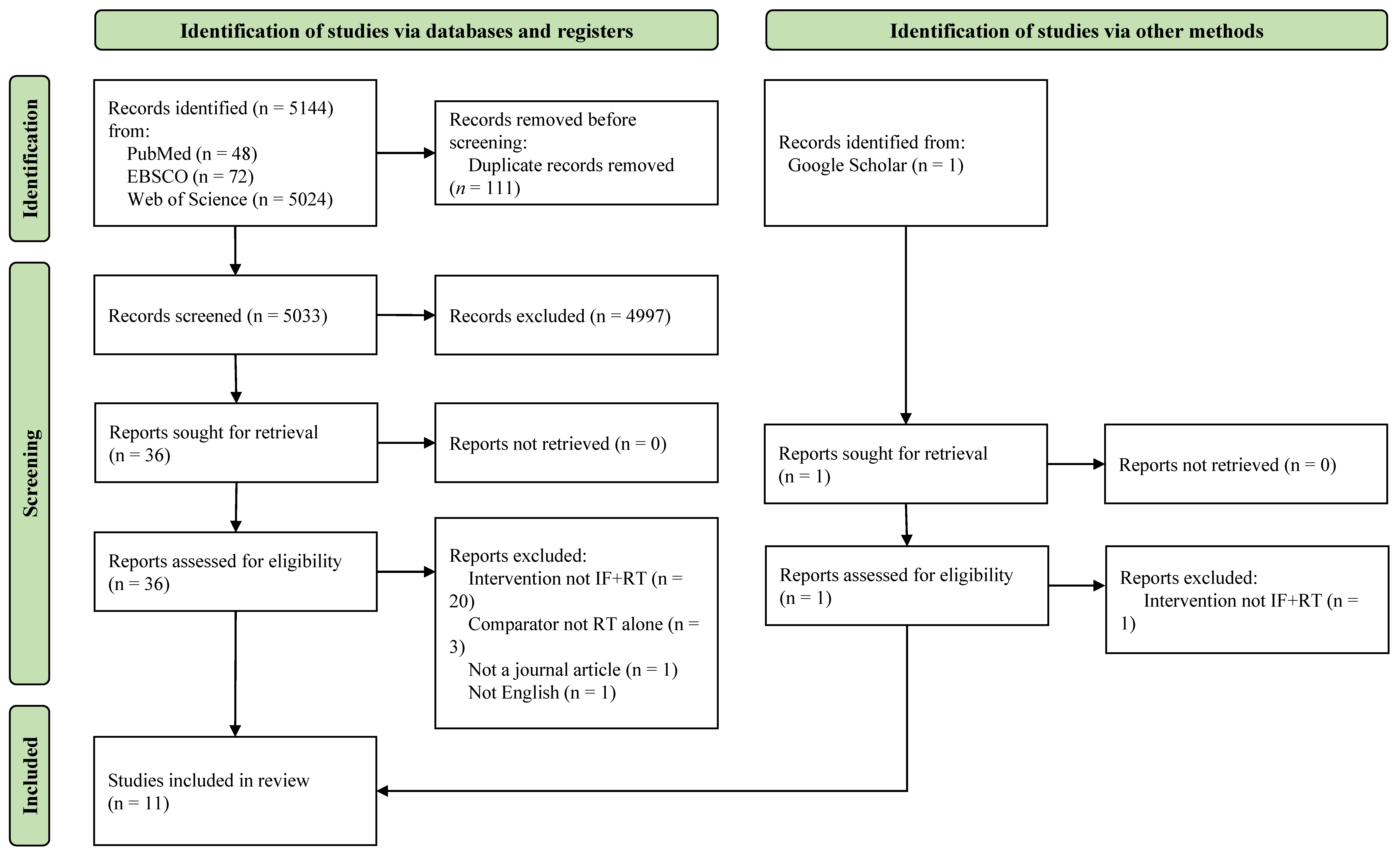


Figure 1 The study selection flow diagram of included and excluded research.


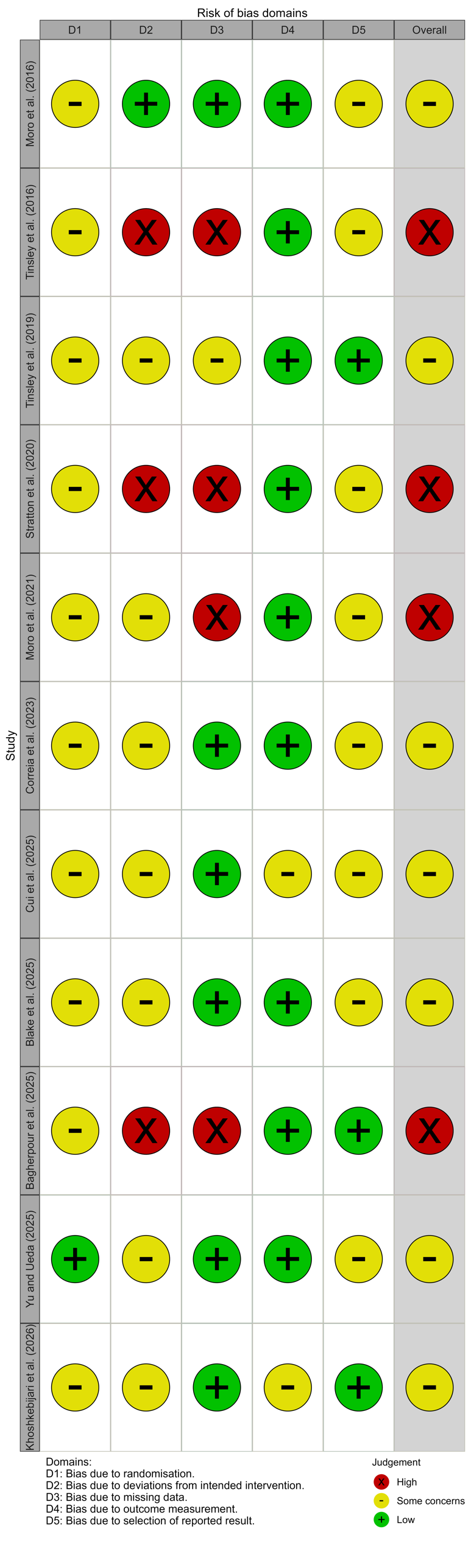


Figure 2 The risk of bias (Green circles represent low risk, yellow circles represent some concerns, and red circles represent high risk).

## Supplementary Tables

Table 1 The specific search strategies for each database.

| Database | Step | Search strategies | Date | Number |
| --- | --- | --- | --- | --- |
| PubMed | #1 | intermittent fasting; time restricted eating; time restricted feeding; alternate day fasting; alternate day modified fasting; 5:2 diet; 5:2 fasting; periodic fasting; feeding window; eating window; fasting mimicking diet | 2026/3/10 | 48 |
|  | #2 | resistance training; resistance exercise; strength training; weight training; muscle strengthening |  |  |
|  | #3 | #1 AND #2 |  |  |
| EBSCO | #1 | AB (intermittent fasting) OR AB (time restricted eating) OR AB (time restricted feeding) OR AB (alternate day fasting) OR AB (alternate day modified fasting) OR AB (5:2 diet) OR AB (5:2 fasting) OR AB (periodic fasting) OR AB (feeding window) OR AB (eating window) OR AB (fasting mimicking diet) | 2026/3/10 | 72 |
|  | #2 | AB (resistance training) OR AB (resistance exercise) OR AB (strength training) OR AB (weight training) OR AB (muscle strengthening) |  |  |
|  | #3 | #1 AND #2 |  |  |
| WOS | #1 | intermittent fasting (Topic) or time restricted eating (Topic) or time restricted feeding (Topic) or alternate day fasting (Topic) or alternate day modified fasting (Topic) or 5:2 diet (Topic) or 5:2 fasting (Topic) or periodic fasting (Topic) or feeding window (Topic) or eating window (Topic) or fasting mimicking diet (Topic) | 2026/3/10 | 5024 |
|  | #2 | resistance training (Topic) or resistance exercise (Topic) or strength training (Topic) or weight training (Topic) or muscle strengthening (Topic) |  |  |
|  | #3 | #1 AND #2 |  |  |

Table 2 The summary of the studies included in the review

| Author | Sample size (sex) | Age (years) | Participant characteristics | Intervention type | Control diet | Control training | Experimental diet | Experimental training |
| --- | --- | --- | --- | --- | --- | --- | --- | --- |
| Moro, Tinsley (1) | 34 (M) | 29.2 ± 3.8 | Resistance-trained men | Chronic, 8 weeks | Eucaloric non-TRE diet | RT, 3 sessions/week; split routine; bench press, incline dumbbell fly, biceps curl, military press, leg press, leg extension, leg curl, wide-grip lat pulldown, reverse-grip lat pulldown, and triceps press-down; 3 sets × 6–8 reps at 85–90% 1RM | Eucaloric 16:8 TRE | Same RT program as control |
| Tinsley, Forsse (2) | 18 (M) | approximately 22–23 | Recreationally active men | Chronic, 8 weeks | Habitual non-TRE diet | RT, 3 sessions/week; upper/lower-body program; bench press, seated row, dumbbell shoulder press, lat pulldown, dumbbell curls, triceps extension, squat or hip sled, lunges, leg curl, leg extension, and calf raise; 4 sets × 8–12 reps to failure | Modified TRE (4-h feeding window, 4 days/week) | Same RT program as control |
| Tinsley, Moore (3) | 40 (F) | approximately 22 | Resistance-trained women | Chronic, 8 weeks | Non-TRE control diet with usual meal timing | RT, 3 sessions/week; alternating upper-body A, lower-body A, upper-body B, and lower-body B sessions; rows, bench press, shoulder press, flyes, curls, triceps extension, deadlift, hip sled, lunges, leg curls, leg extensions, back squat, stiff-leg deadlift, and inverted rows; W0–W4: mostly 4 × 8–12; W4–W8: major lifts progressed to 5 × 6–8 | 16:8 TRE | Same RT program as control |
| Stratton, Tinsley (4) | 26 (M) | approximately 22–23 | Recreationally active men | Chronic, 4 weeks | Hypocaloric non-TRE diet | RT, 3 sessions/week; full-body program; bench press, leg press, row, shoulder press, leg extension, hamstring curl, and arm exercises; periodized loading | Hypocaloric 16:8 TRE | Same RT program as control |
| Moro, Tinsley (5) | 20 (M) | NR | Resistance-trained men | Chronic, 12 months | Eucaloric non-TRE diet | RT, 3 sessions/week; long-term RT continued after the initial supervised phase; intensity fluctuated between 75% and 90% 1RM to alternate strength and hypertrophy cycles | Eucaloric 16:8 TRE | Same RT program as control |
| Correia, Santos (6) | 18 (M) | 23.7 ± 2.6 | Resistance-trained men | Chronic crossover trial, 4 weeks per condition, 2-week washout | Habitual non-TRE diet | RT, 3 sessions/week; leg press, chest press, leg curl, lat pulldown, leg extension, shoulder press, and abdominal crunches; 4 sets of maximal repetitions at 85% 1RM | 8-h TRE | Same RT program as control |
| Cui, Sun (7) | 54 (36 F, 18 M) | approximately 20 | Young adults with overweight/obesity | Chronic, 8 weeks | Habitual non-TRE diet | RT, 3 sessions/week; bench press, high pull-down, dumbbell curls, leg press, seated leg curls, and leg extension; 4 sets × 8–12 reps at 60–70% 1RM | 10-h TRE | Same RT program as control |
| Blake, Hamane (8) | 17 (10 M, 7 F) | approximately 23–26 | Resistance-trained men and women | Chronic, 8 weeks | Hypercaloric non-TRE diet | RT, 4 sessions/week; A-B-A-B program. Workout A: Romanian deadlift, leg extension, barbell bench press, neutral-grip pulldown, seated calf raise, and cable lateral raise. Workout B: high-bar squat, lying leg curl, barbell overhead press, seated row, cable chest fly, triceps pushdown, and seated dumbbell curl | Hypercaloric 16:8 TRE | Same RT program as control |
| Bagherpour, Arazi (9) | 32 (F) | 23.0 ± 2.6 | Sedentary women with overweight | Chronic, 8 weeks | Hypocaloric non-TRE diet | RT, 3 sessions/week; chest press, lat pulldown, biceps curls, leg press, leg extension, leg flexion, plank, and back extensions; 3 sets × 8–10 reps, progressing from 65% to 80% 1RM | Hypocaloric TRE | Same RT program as control |
| Yu and Ueda (10) | 24 (F) | 22.1–24.1 | Healthy young women with limited/no regular RT experience | Chronic, 8 weeks | Non-TRE diet (08:00–20:00 eating window) | RT, 3 sessions/week; knee-supported push-ups; 4 sets × 10 reps | eTRE (08:00–14:00) or dTRE (12:00–18:00) | Same RT program as control |
| Farahmand Khoshkebijari, Ebrahimi (11) | 20 (M) | 24.4–25.5 | Sedentary men with obesity | Chronic, 8 weeks | Habitual non-TRE diet | RT, 3 sessions/week; bench press, deadlift, back squat, hamstring curl, standing dumbbell shoulder press, standing barbell curl, and overhead triceps extension; mainly 3–4 sets × 10 reps at 70% 1RM | 4:3 IF | Same RT program as control |

Note: M, male; F, female; RT, resistance training; TRE, time-restricted eating; TRF, time-restricted feeding; IF, intermittent fasting; NR, not reported; 1RM, one-repetition maximum; reps, repetitions; W0–W4, weeks 0 to 4; W4–W8, weeks 4 to 8; h, hours.

Table 3 Studies and comparisons retained or excluded in sensitivity analyses.

| **Outcome domain** | **Sensitivity analysis** | **Retained studies/comparisons** | **Excluded studies/comparisons** | **Notes / pooled result** |
| --- | --- | --- | --- | --- |
| Primary outcomes | High-confidence-only analysis | Moro, Tinsley (1) (TRF vs ND); Tinsley, Forsse (2) (TRF vs ND); Moro, Tinsley (5) (TRE vs ND); Bagherpour, Arazi (9) (THP vs HP; TRP vs RP); Yu and Ueda (10) (dTRE vs Control; eTRE vs Control). Retained: k = 18, study clusters = 5, comparisons = 7. | Tinsley, Moore (3) (TRF vs CD): bench press 1RM, bench press repetitions to failure, leg press 1RM, and leg press repetitions to failure. Excluded: k = 4. | Excluded because extraction confidence was moderate. Pooled result: g = 0.19 [0.01, 0.38], p = 0.042. |
| Primary outcomes | Excluding shared-control comparisons | All eligible primary-outcome comparisons except the shared-control comparisons from Yu and Ueda (10). Retained: k = 20, study clusters = 5, comparisons = 6. | Yu and Ueda (10) (dTRE vs Control; eTRE vs Control): push-up endurance. Excluded: k = 2. | Excluded because both intervention arms shared the same control group. Pooled result: g = 0.10 [-0.26, 0.46], p = 0.481. |
| Primary outcomes | Varying assumed pre-post correlation | All eligible primary-outcome comparisons were retained: Moro, Tinsley (1); Tinsley, Forsse (2); Tinsley, Moore (3); Moro, Tinsley (5); Bagherpour, Arazi (9); Yu and Ueda (10). Retained: k = 22, study clusters = 6, comparisons = 8. | None. | The assumed pre-post correlation used to estimate change-score SDs was varied from r = 0.30 to 0.90. Pooled effects remained small and non-significant (g = 0.05 to 0.16). |
| Secondary hypertrophy-related outcomes | High-confidence-only analysis | Moro, Tinsley (1) (TRF vs ND); Stratton, Tinsley (4) (TRF vs ND); Moro, Tinsley (5) (TRE vs ND); Yu and Ueda (10) (dTRE vs Control; eTRE vs Control). Retained: k = 12, study clusters = 4, comparisons = 5. | Tinsley, Moore (3) (TRF vs CD): elbow flexor muscle thickness and knee extensor muscle thickness. Excluded: k = 2. | Excluded because extraction confidence was moderate. Pooled result: g = 0.08 [-0.27, 0.43], p = 0.447. |
| Secondary hypertrophy-related outcomes | Excluding shared-control comparisons | All eligible secondary hypertrophy-related comparisons except the shared-control comparisons from Yu and Ueda (10). Retained: k = 12, study clusters = 4, comparisons = 4. | Yu and Ueda (10) (dTRE vs Control; eTRE vs Control): triceps muscle thickness. Excluded: k = 2. | Excluded because both intervention arms shared the same control group. Pooled result: g = 0.09 [-0.22, 0.41], p = 0.358. |
| Secondary hypertrophy-related outcomes | Varying assumed pre-post correlation | All eligible secondary hypertrophy-related comparisons were retained: Moro, Tinsley (1); Tinsley, Moore (3); Stratton, Tinsley (4); Moro, Tinsley (5); Yu and Ueda (10). Retained: k = 14, study clusters = 5, comparisons = 6. | None. | The assumed pre-post correlation used to estimate change-score SDs was varied from r = 0.30 to 0.90. Pooled effects remained small and non-significant (g = 0.02 to 0.08). |

**Note:** IF, intermittent fasting; RT, resistance training; TRE, time-restricted eating; TRF, time-restricted feeding; dTRE, delayed time-restricted eating; eTRE, early time-restricted eating; ND, normal diet; CD, control diet; HP, high protein; RP, regular protein; THP, time-restricted eating plus high protein; TRP, time-restricted eating plus regular protein; 1RM, one-repetition maximum; SD, standard deviation; k, number of effect sizes.

Table 4 Protein and carbohydrate intake reporting across included studies.

| **Author** | **Protein intake reporting** | **Protein interpretation** | **Carbohydrate intake reporting** | **Carbohydrate interpretation** |
| --- | --- | --- | --- | --- |
| Moro, Tinsley (1) | Reported during the intervention. TRF: 1.93 ± 0.3 g/kg/day; ND: 1.89 ± 0.4 g/kg/day. No significant differences were reported between or within groups. | Protein intake was similar between groups and likely sufficient to support RT adaptations. | Reported during the intervention. TRF: 1400.3 ± 118.8 kcal/day; ND: 1609.2 ± 201.5 kcal/day. No significant differences were reported. | Carbohydrate intake was broadly comparable between groups. |
| Tinsley, Forsse (2) | Reported from 4-day dietary records. Weekly protein intake was numerically lower in RT-TRF at weeks 4 and 8 than in RT-ND, but the group effect was not significant. | Protein intake may have been lower and potentially suboptimal in the RT-TRF group, which should be considered when interpreting lean-mass and performance adaptations. | Reported from 4-day dietary records. Weekly carbohydrate intake was lower in RT-TRF than in RT-ND (group effect significant). | Lower carbohydrate and energy intake in RT-TRF may have influenced training performance, recovery, or adaptations. |
| Tinsley, Moore (3) | Reported. Energy and protein intake did not differ between groups; protein intake was approximately 1.6 g/kg/day during the intervention. | Protein intake was similar between groups and likely sufficient. | Reported. Carbohydrate and fat intake generally did not change during the intervention. | No clear evidence of a major carbohydrate imbalance between groups. |
| Stratton, Tinsley (4) | Reported. TRF: 1.83 ± 0.10 g/kg/day; ND: 1.83 ± 0.07 g/kg/day (p = 0.956). | Protein intake was closely matched and likely sufficient. | Reported. TRF: 2.28 ± 0.71 g/kg/day; ND: 2.25 ± 0.51 g/kg/day (p = 0.889). | Carbohydrate intake was closely matched between groups. |
| Moro, Tinsley (5) | Reported across 12 months. At 12 months, TRE: 1.92 ± 0.13 g/kg/day; ND: 1.93 ± 0.13 g/kg/day; time-by-diet interaction was not significant. | Protein intake was similar between groups and likely sufficient. | Reported across 12 months. At 12 months, carbohydrate intake was lower in TRE than ND (1374.5 ± 132.5 vs 1606.2 ± 152.6 kcal/day; time-by-diet interaction significant). | Lower carbohydrate and total energy intake in TRE may have contributed to some long-term between-group differences. |
| Correia, Santos (6) | Reported from baseline dietary records before each condition. TRE: 2.0 ± 0.5 g/kg/day; non-TRE: 1.9 ± 0.6 g/kg/day (p = 0.45). | Protein intake appeared similar and sufficient before each condition, but intake during the intervention was not fully verified. | Reported from baseline dietary records. Carbohydrate percentage was similar between conditions (TRE: 44.1 ± 6.0%; non-TRE: 44.3 ± 7.1%; p = 0.87). | Baseline carbohydrate intake appeared similar, but changes during the intervention were unclear. |
| Cui, Sun (7) | Not recorded. The authors stated that calorie intake, macronutrients, and protein intake data were not recorded. | Protein adequacy and between-group similarity could not be verified. | Not recorded. | The potential influence of carbohydrate intake could not be evaluated. |
| Blake, Hamane (8) | Reported/prescribed. Both groups consumed a hypercaloric high-protein diet (2.2 g/kg/day), and protein intake did not differ statistically between groups. | Protein intake was similar between groups and likely sufficient. | Reported. Calorie, carbohydrate, fat, and protein intake did not differ statistically between groups. | Carbohydrate intake was comparable between groups. |
| Bagherpour, Arazi (9) | Reported/prescribed by diet group. THP and HP targeted 1.6 g/kg/day; TRP and RP targeted 0.8 g/kg/day. | Protein intake was matched within the relevant TRE vs non-TRE protein comparisons; high-protein arms were likely sufficient, whereas regular-protein arms were lower. | Reported/prescribed. Both high- and regular-protein diets provided approximately 50% of energy from carbohydrate. | Carbohydrate intake was broadly comparable within the relevant comparisons. |
| Yu and Ueda (10) | Partly reported. Participants were required to consume at least 1.2 g/kg/day of protein, monitored using dietary photo logs and subjective descriptions; precise daily intake was not reported. | Protein adequacy and between-group similarity were only partly verifiable. | Precise carbohydrate intake was not reported. | The potential influence of carbohydrate intake could not be evaluated. |
| Farahmand Khoshkebijari, Ebrahimi (11) | Not reported. The study noted that dietary intake was difficult to match and that future studies should consider food recalls. | Protein adequacy and between-group similarity could not be verified. | Not reported. | The potential influence of carbohydrate intake could not be evaluated. |

Note: RT, resistance training; TRE, time-restricted eating; TRF, time-restricted feeding; ND, normal diet; THP, time-restricted eating plus high protein; TRP, time-restricted eating plus regular protein; HP, high protein; RP, regular protein. Protein sufficiency was interpreted cautiously using commonly applied RT nutrition targets; no subgroup or meta-regression analysis was performed based on protein or carbohydrate intake.

References

1. Moro T, Tinsley G, Bianco A, Marcolin G, Pacelli QF, Battaglia G, et al. Effects of Eight Weeks of Time-Restricted Feeding (16/8) on Basal Metabolism, Maximal Strength, Body Composition, Inflammation, and Cardiovascular Risk Factors in Resistance-Trained Males. *J Transl Med* (2016) 14(1):290. Epub 20161013. doi: 10.1186/s12967-016-1044-0.

2. Tinsley GM, Forsse JS, Butler NK, Paoli A, Bane AA, La Bounty PM, et al. Time-Restricted Feeding in Young Men Performing Resistance Training: A randomized Controlled Trial. *Eur J Sport Sci* (2016) 17(2):200-7. Epub 20160822. doi: 10.1080/17461391.2016.1223173.

3. Tinsley GM, Moore ML, Graybeal AJ, Paoli A, Kim Y, Gonzales JU, et al. Time-Restricted Feeding Plus Resistance Training in Active Females: A Randomized Trial. *Am J Clin Nutr* (2019) 110(3):628-40. doi: 10.1093/ajcn/nqz126.

4. Stratton MT, Tinsley GM, Alesi MG, Hester GM, Olmos AA, Serafini PR, et al. Four Weeks of Time-Restricted Feeding Combined with Resistance Training Does Not Differentially Influence Measures of Body Composition, Muscle Performance, Resting Energy Expenditure, and Blood Biomarkers. *Nutrients* (2020) 12(4). Epub 20200417. doi: 10.3390/nu12041126.

5. Moro T, Tinsley G, Pacelli FQ, Rcolin G, Bianco A, Paoli A. Twelve Months of Time-Restricted Eating and Resistance Training Improves Inflammatory Markers and Cardiometabolic Risk Factors. *Medicine & Science in Sports & Exercise* (2021) 53(12):2577-85. doi: 10.1249/MSS.0000000000002738.

6. Correia JM, Santos PDG, Pezarat-Correia P, Minderico CS, Infante J, Mendonca GV. Effect of Time-Restricted Eating and Resistance Training on High-Speed Strength and Body Composition. *Nutrients* (2023) 15(2). doi: 10.3390/nu15020285.

7. Cui T, Sun Y, Ye W, Liu Y, Korivi M. Efficacy of Time Restricted Eating and Resistance Training on Body Composition and Mood Profiles among Young Adults with Overweight/Obesity: A Randomized Controlled Trial. *Journal of the International Society of Sports Nutrition* (2025) 22(1):2481127. doi: 10.1080/15502783.2025.2481127.

8. Blake DT, Hamane C, Pacheco C, Henselmans M, Tinsley GM, Costa P, et al. Hypercaloric 16:8 Time-Restricted Eating during 8 Weeks of Resistance Exercise in Well-Trained Men and Women. *Journal of the International Society of Sports Nutrition* (2025) 22(1):2492184. doi: 10.1080/15502783.2025.2492184.

9. Bagherpour F, Arazi H, Rajabi H, Campbell BI. High-Protein Time-Restricted Eating Alongside Resistance Training Reduces Adipose Tissue While Preserving Fat-Free Mass in Women with Overweight: A Randomized Controlled Trial. *International Journal of Sport Nutrition & Exercise Metabolism* (2025) 35(6):493-509. doi: 10.1123/ijsnem.2025-0010.

10. Yu Z, Ueda T. Early Time-Restricted Eating Improves Weight Loss While Preserving Muscle: An 8-Week Trial in Young Women. *Nutrients* (2025) 17(6). doi: 10.3390/nu17061022.

11. Farahmand Khoshkebijari A, Ebrahimi M, Jorbonian A, Kaushik S. Intermittent Fasting May Enhance Resistance Training Effects on the Body Composition of Obese Males, without Affecting Muscular Strength and Anabolic Index. *Journal of Obesity* (2026) 2026:1-8. doi: 10.1155/jobe/6409069.
